# Robert (Bob) Bluglass, CBE, DPM, FRCPsych., FRCP (London)

**DOI:** 10.1192/bjb.2023.37

**Published:** 2024-02

**Authors:** Femi Oyebode

Formerly Emeritus Professor of Psychiatry, University of Birmingham, UK

**Figure:**
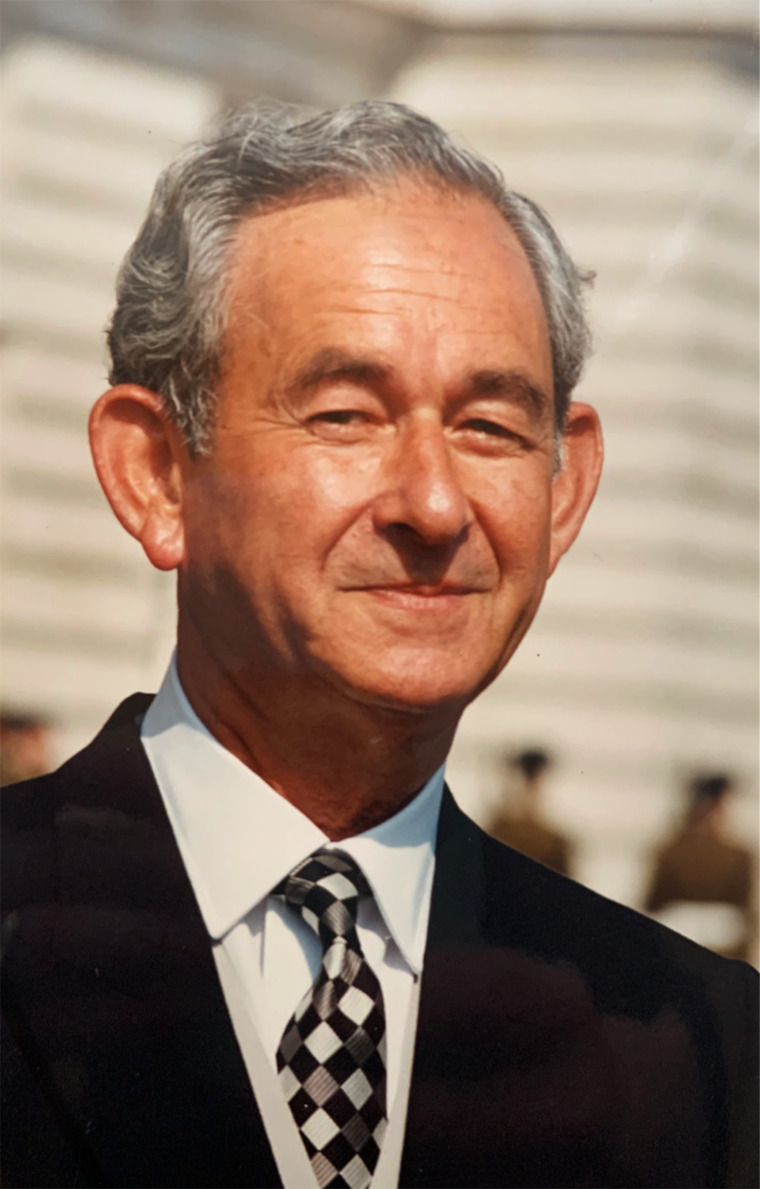

Robert Bluglass, who died aged 91 on 1 August 2022, was a pioneer of modern forensic psychiatry in the UK. He was one of the first consultants in forensic psychiatry, establishing the Midland Centre for Forensic Psychiatry at All Saints’ Hospital Birmingham as the first regional forensic psychiatry service. He became the Clinical Director of the Reaside Clinic when it opened in October 1987 as the regional secure unit for the West Midlands region. Reaside Clinic was and remains a centre of excellence, providing a modern forensic psychiatry service. Bluglass remarked in his valedictory address that ‘it took fourteen years of monthly project team meetings and planning conferences to design and build the Reaside Clinic’ and he credited the 1975 Butler Report (*Report of the Committee on Mentally Abnormal Offenders*) for its vision and considerable influence on the development of secure units.

Jointly with Paul Bowden, Bluglass edited *Principles and Practice of Forensic Psychiatry*, which was published in 1990. This was a monumental work of 1500 pages, with contributions from 150 psychiatrists and scholars. It is widely regarded as the first comprehensive textbook of the field. In the Preface, Bluglass and Bowden wrote ‘The field of operation is the overlap, interface and interaction of psychiatry and the law in all its aspects; criminal behaviour, civil litigation, family law, the diagnosis, care and treatment of psychiatric patients where the disorder is associated with abnormalities of behaviour, legislation and numerous other problems such as the management of violence and the study of sexual deviance’ (p. vii). The scope of the book was wide, with chapters on criminology, responsibility, the relationship between mental disorder and crime, personality disorder, and violent behaviour and its management. Bluglass himself contributed seven chapters.

Jointly with Peter Fallon QC, Brian Edwards and Granville Daniels, Bluglass presented the *Report of the Committee of Inquiry into the Personality Disorder Unit, Ashworth Special Hospital* in January 1999 to the Secretary of State for Health, Frank Dobson. The inquiry had arisen in the context of the allegations of a former patient, Steven Daggett, about the misuse of substances and alcohol, financial irregularities, possible paedophilic activity and the availability of pornographic material on the Personality Disorder Unit. The report concluded that these allegations were largely accurate. The Report was damning of Ashworth Special Hospital and concluded that the Personality Disorder Unit was a deeply flawed creation and that there were gross failures in the care and management of a large group of patients with severe personality disorder. The Report made 58 recommendations about the hospital, its security and the wider problems of dealing with violent criminals with personality disorders. It suggested that the Personality Disorder Unit, where the activities took place, should be managed in smaller units of no more than 8 to 12 patients. It recommended that the Department of Health and the Home Office develop new, regional special units for people with severe personality disorders. The report also called for a change to the Mental Health Act 1983 which would remove the classification ‘psychopathic disorder’ and replace it with ‘personality disorder’. Mr Dobson, rejecting the inquiry's proposal to close Ashworth, said that the problem lay in management, not in the bricks and mortar, and argued that there was no practical alternative in the short term to the three special hospitals in the UK: Ashworth, Rampton and Broadmoor.

Robert Bluglass was born in Golders Green, North London, in 1930, the elder of two children. His father Henry was the chief accountant at M. Samuel & Co merchant bank. His mother Faye (née Griew) was an artist. The family was evacuated to Warwickshire during the Second World War and Bluglass remained as a boarder at Warwick School when the family returned to London. He studied medicine at the University of St Andrews and completed his house jobs at Dundee Royal Mental Hospital, where he later trained with Professor Sir Ivor Batchelor, whom he found inspiring even if demanding. Bluglass completed his MD on the prevalence of psychiatric disorders among the inmates of Perth prison and investigated the value of establishing a forensic psychiatry service in the east of Scotland. He was appointed consultant psychiatrist in 1967 and Professor of Psychiatry at the University of Birmingham in 1979.

Bluglass was closely involved in the revision of the Mental Health Act 1959 that resulted in the Mental Health Act 1983. He commuted weekly to the House of Commons and this work resulted in *A Guide to the Mental Health Act 1983*, published in 1983. He was Vice President of the Royal College of Psychiatrists from 1983 to 1985. He was awarded a CBE in 1995.

He enjoyed watercolour painting and visits to Italy in his retirement. He inherited a keen artistic eye from his mother, Faye. Each year he and his wife would join a group of medical painters in Tuscany.

He is survived by his wife, Kerry (née Montgomery), who is a psychiatrist and former senior clinical lecturer at the University of Birmingham, a son, Charles, a daughter, Amanda, and two grandchildren, Luke and Maya.

